# A Single-Health System Case Series of New-Onset CNS Inflammatory Disorders Temporally Associated With mRNA-Based SARS-CoV-2 Vaccines

**DOI:** 10.3389/fneur.2022.796882

**Published:** 2022-02-24

**Authors:** Ahmad A. Ballout, Anna Babaie, Michael Kolesnik, Jian Yi Li, Natasha Hameed, Glenn Waldman, Frasat Chaudhry, Sami Saba, Asaff Harel, Souhel Najjar

**Affiliations:** ^1^Department of Neurology, Donald and Barbara Zucker School of Medicine at Hofstra/Northwell, Hempstead, NY, United States; ^2^Pathology, Northwell Health, Donald and Barbara Zucker School of Medicine at Hofstra/Northwell, Hempstead, NY, United States

**Keywords:** ADEM, mRNA vaccine, multiple sclerosis, CNS inflammation, COVID-19 vaccination

## Abstract

**Background:**

Since 2020, over 250 million doses of mRNA-based SARS-CoV-2 vaccines have been administered in the United States and hundreds of millions worldwide between the Pfizer-BioNTech and Moderna SARS-CoV-2 vaccines. To date, there have been rare reports associating mRNA-based SARS-CoV-2 vaccines with episodes of inflammatory and autoimmune CNS disorders. We report a case series of five patients with new-onset neurological disorders of inflammatory or immunological origin temporally associated with these vaccines.

**Methods:**

A case-series of five patients within a single 23-hospital health system who developed new-onset CNS inflammatory disease within 2 weeks of receiving a dose of an mRNA-based SARS-CoV-2 vaccine.

**Results:**

Five cases of post-vaccination CNS disorders of immune origin (fatal ADEM; *n* = 1, new-onset NMOSD; *n* = 2, new-clinical onset MS-like syndrome but with preexisting clinically silent mild demyelination; *n* = 1, meningoencephalitis; *n* = 1) observed within 2 weeks of inoculation with either the first or second dose of mRNA-based SARS-CoV-2 vaccines (Moderna = 3, Pfizer = 2).

**Discussion:**

To our knowledge, these are among the emerging cases of CNS adverse events of immunological or inflammatory origin. These findings should be interpreted with great caution as they neither prove a mechanistic link nor imply a potential long-term increased risk in post-vaccination CNS autoimmunity. Larger prospective studies assessing the potential association between mRNA-based vaccination and the development of neurological adverse events of suspected immune origin, particularly among those with underlying CNS or systemic autoimmune disorders, are needed. The use of mRNA-based SARS-CoV-2 vaccines should continue to be strongly encouraged given their high efficacy in overcoming this pandemic.

## Introduction

Although several vaccines have been temporally associated with central nervous system (CNS) inflammatory events, including monophasic disorders like acute disseminated encephalomyelitis (ADEM) (1), or exacerbations of chronic conditions like neuromyelitis optica spectrum disorder (NMOSD) (2, 3), and multiple sclerosis (MS) (4, 5), there have not been any reports mechanistically linking mRNA-based SAR-CoV-2 vaccines to a higher rate of inflammatory or autoimmune CNS disease.

ADEM is a rare, typically monophasic, CNS inflammatory disease that commonly occurs after a viral infection, including instances associated with SARS-CoV-2 infection (6, 7). Due to a presumed process involving molecular mimicry, ADEM has been temporally associated with various vaccines, such as rabies, hepatitis B, measles, mumps, rubella, pertussis, diphtheria, varicella, influenza, Japanese encephalitis, and polio vaccines, with an estimated incidence of 0.1–0.2 per 100,000, often occurring between 1 and 3 weeks following vaccine administration (1).

NMOSD is a chronic CNS inflammatory disorder that predominantly involves the spinal cord and optic nerves. NMOSD relapses have been associated with multiple infections, including SARS-CoV-2 infections (8, 9), and several case reports have suggested a temporal association between NMOSD relapses and various vaccines such as the influenza, tetanus diphtheria and pertussis, human papilloma virus, pneumococcal, hepatitis A, hepatitis B, typhoid, yellow fever, and Japanese encephalitis vaccines (2, 3). In contrast to NMOSD, vaccination does not generally increase the short-term risk of relapse in MS other than a few cases reported following the yellow fever vaccine (4, 5). Since post-marketing vaccination against SARS-CoV-2 began in 2020, there have been isolated reports of CNS autoimmunity, including one case of ADEM following the inactivated BBIBP-CorV SARS-CoV-2 vaccine in China (10). Recently, several MS exacerbations (new-onset MS; *n* = 2, exacerbation of clinically stable MS; *n* = 4) as well as one *de-novo* NMOSD diagnosis were reported among the recipients of SARS-CoV-2 mRNA vaccination (11–13). In an international study of 27 cases of new-onset or relapse of immune mediated disease following SARS-CoV-2 vaccination using various platforms, there was one case of new-onset MS following the administration of the Pfizer-BioNtech vaccine (14). Three cases of antibody-negative “possible” autoimmune encephalitis were reported after the administration of the ChAdOx1 nCoV-19 vector-based vaccine, including a case of opsoclonus-myoclonus syndrome (15). We report five separate cases of CNS autoimmunity and inflammatory pathologies that occurred in previously healthy individuals shortly following the administration of mRNA-based SARS-CoV-2 vaccines at a single health system in the greater New York City area.

## Materials and Methods

This is a case-series of five patients within a single 23-hospital health system who developed new-onset CNS inflammatory disease within 2 weeks of receiving a dose of an mRNA-based SARS-CoV-2 vaccine. Since this was a case series limited to patients who were diagnosed and treated by the study authors, rather than a systemic review of all patients within the health system who may have developed new-onset CNS inflammatory disease within a pre-specified 2-week period of receiving the vaccine, there may be other undetected cases not included in this study. This report was approved by the Feinstein Institutes for Medical Research IRB (approval # 20-0600). Written consent was obtained from all of the patients or their families. Anonymized data not published within this article is available upon request.

## Case Presentations

### Case #1: ADEM

An 81-year-old man with no relevant neurological history presented to the emergency department (ED) with rapid-onset acute change in mental status with severe encephalopathy noted about 13 days following the administration of the first dose of the Moderna SARS-CoV-2 vaccine. It was also preceded by prodromal symptoms of viral-like illness marked by several days of low-grade fever, fatigue, and myalgia. He had a fever of 102°F without skin rashes or nuchal rigidity. Neurological exam revealed minimal response to noxious stimuli, right gaze preference, minimal horizontal eye movements upon oculocephalic testing, absent pupillary response to light, absent right corneal reflex, diffuse hypertonicity, and extensor plantar responses bilaterally. Head CT and CT angiogram of the head and neck were unremarkable. Serologies demonstrated mild leukocytosis with WBC count of 12.5 K/μL (reference range 3.8–10.5 K/μL), Erythrocyte Sedimentation Rate (ESR) of 86 mm/hr (reference range 1–15 mm/h) and C-Reactive Protein (CRP) of 10.8 mg/L (reference range <4.9 mg/L). Cerebrospinal Fluid (CSF) analysis demonstrated an opening pressure of 26 cmH2O, glucose of 69 mg/dL (reference range 40–70 mg/dL), protein of 45 mg/dL (reference range 15–45 mg/dL), and WBC count of 3 cells/μL (reference range 0–5 cells/μL). Infectious workup was negative. It included urine culture, urine legionella, respiratory virus panel PCR, encompassing influenza, parainfluenza, adenovirus, respiratory syncytial virus, Chlamydia pneumoniae, Mycoplasma pneumoniae, and enterovirus. Nasopharyngeal COVID-19 PCR and SARS-CoV-2 antibodies against nucleocapsid protein were negative (antibody formation to spike protein was not performed). Blood cultures drawn on admission (day 1) and twice afterwards (day 5 and 10) were without growth. CSF infectious workup was negative which included HSV PCR, bacterial cultures, gram stain, cryptococcal antigen, VDRL titer, fungal culture, and listeria antibody. Serologies for blastomycoses, cryptococcus, coccidioidies, and galactomannan were negative. HIV testing was negative. QuantiFERON testing was performed twice and was indeterminate (however titers were low), imaging was negative for tuberculoma, and CSF glucose and total nucleated cell count was normal lowering suspicion of tuberculosis meningitis. His neurological condition deteriorated rapidly to a comatose state within the subsequent 24 h and required emergent intubation. He continued to spike fevers despite broad-spectrum antibiotics and a negative infectious workup. On day 10, patient was found to be positive for clostridium difficile and was started on oral vancomycin for which he completed a course.

**Figure 1:**
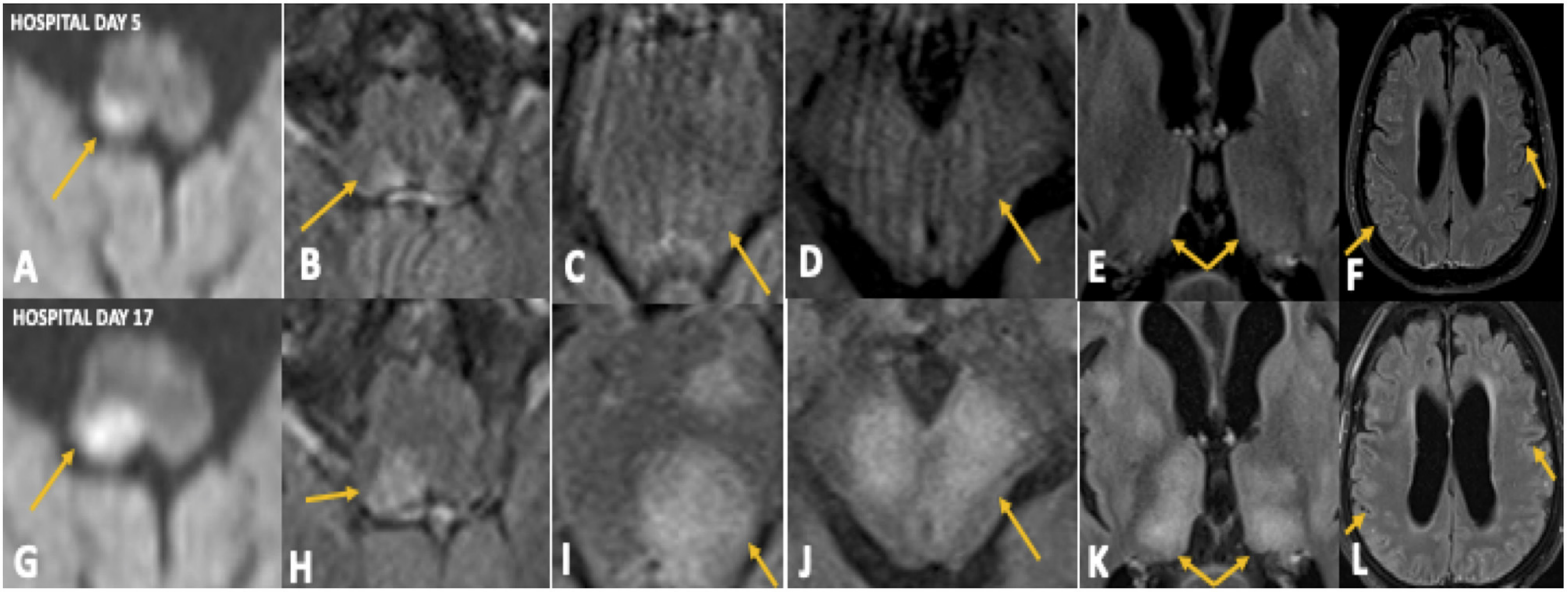
Case 1: Initial MRI brain on hospital day 5 demonstrated diffusion restriction in the right dorsal medulla **(A)** with corresponding T2 FlAIR hyperintensity **(B)**, faint T2 hyperintensities in the left pons **(C)**, midbrain **(D)**, and thalamus **(E)**, and minimal T2 sulcal hyperintensity without contrast enhancement **(F)**. Repeat MRI brain on hospital day 17 showing significant progression of these abnormalities **(G–L)**.

**Figure 2:**
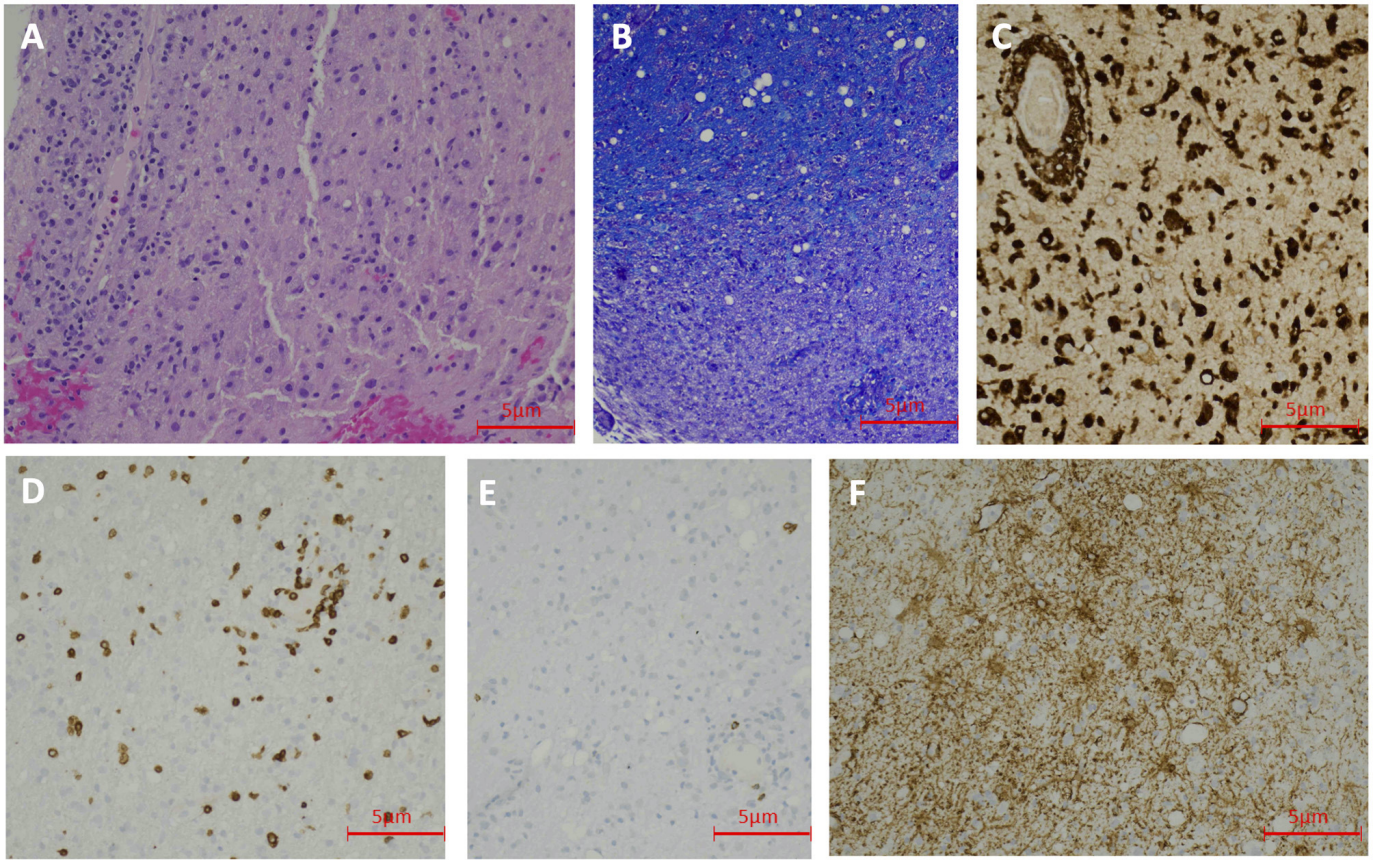
Case 1 Pathology: Hematoxylin and Eosin staining shows lymphohistocytic infiltrate in the brain tissue (200 X) **(A)**. LFB staining demonstrates demyelination (200 X) **(B)**. CD68 immunohistochemical stain shows numerous histiocytes (200 X) **(C)**. CD3 immunohistochemical stain reveals scattered T-lymphocytes (200 X) **(D)**. CD20 immunohistochemical stain demonstrate rare B-lymphocytes (200 X) **(E)**. GFAP immunohistochemical stain shows reactive astrocytes (200 X) **(F)**.

On hospital Day 5, brain MRI with gadolinium showed a diffusion restricting lesion involving the right dorsal medulla with corresponding T2 FlAIR hyperintensity (Figures 1A,B), very faint left pontine, midbrain, and thalamic T2 FlAIR hyperintensity (Figures 1C–E), and minimal T2 sulcal hyperintensity without apparent enhancement (Figure 1F) suggestive of a possible inflammatory or infectious process. On hospital day 9, repeat CSF analysis revealed a mild lymphocytic pleocytosis with a WBC count of 11 cells/μL and protein of 52 mg/dL. A CSF autoimmune encephalitis panel (Mayo Laboratories ID:ENC2) was negative. The patient was started on a 5-day trial of intravenous immunoglobulin (IVIG) for presumed CNS inflammatory disorder without clinical improvement. A third CSF assay completed on hospital day 12 revealed pleocytosis of 69 cells/μL with 83% lymphocytic predominance, protein of 45 mg/dL, and significantly elevated myelin basic protein (MBP) >167.0 ng/mL (reference range 0–6.0 ng/mL). CSF anti-Myelin Oligodendrocyte Glycoprotein (anti-MOG) antibody was negative. A right frontal lobe biopsy was performed on hospital day 16, including leptomeninges and cortex, and showed an acute inflammatory demyelinating process (Figures 2A–F). Stains for infectious agents were negative, including bacterial, fungal, and viral sources (including JC virus). Repeated Brain MRI with gadolinium on hospital day 17 demonstrated multiple, non-enhancing, T2 hyperintense lesions involving bilateral frontoparietal lobes, lentiform nuclei, thalami, cerebral peduncles, pons, and right posterior medulla (Figures 1G–L). The clinical and neuroradiological findings were deemed most consistent with ADEM.

The patient was treated with high-dose IV methylprednisolone, IVIG therapy, and later plasmapheresis for a presumed diagnosis of ADEM, after a reasonable exclusion of alternative etiologies, without a clinical response. He died on hospital day 26 due to hemorrhagic shock of probable gastrointestinal origin.

### Case #2: New-Onset Neuromyelitis Optica Spectrum Disorder

A 63-year-old Guyanese woman with hypothyroidism and hyperlipidemia presented with a 2-week history of severe back pain, rapidly progressive paraparesis, and urinary retention. The onset of neurological symptoms was about 1 week following the first dose of the Pfizer-BioNTech vaccine. General examination was unremarkable with normal vital signs. She was found to have leukopenia (2.55 K/μL). Neurological examination revealed moderate paraparesis of the lower extremities, mild weakness of the right arm, and reduced right facial sensation. The remainder of the segmental neurological examination was unremarkable.

**Figure 3:**
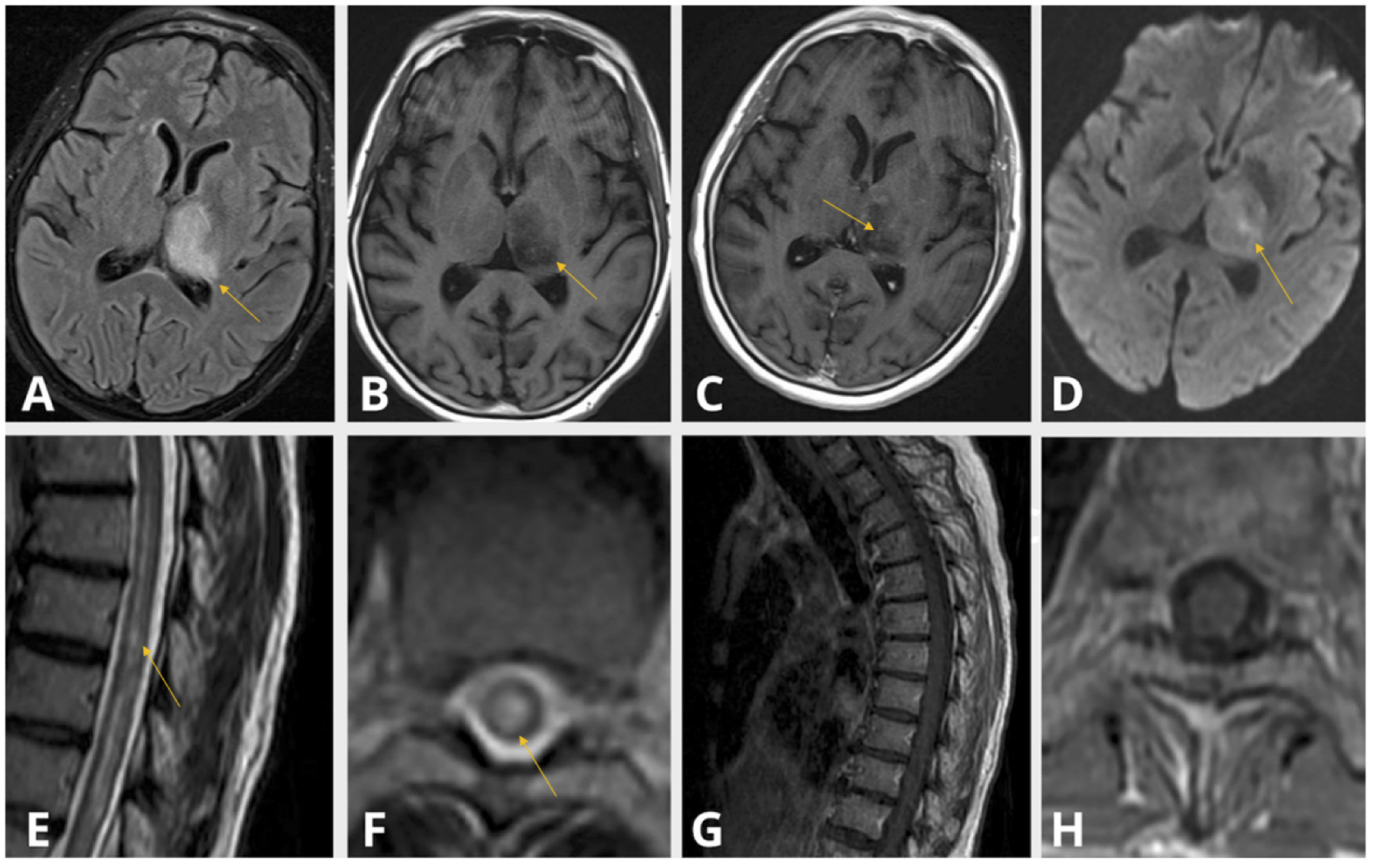
Case 2: Thalamic T2 FlAIR hyperintensity **(A)**, T1 shortening **(B)** with subtle contrast enhancement **(C)** and diffusion restriction **(D)**. Centrally located T2 hyperintensity spanning the length of the thoracic cord **(E,F)** without evidence of contrast enhancement **(G,H)**.

**Figure 4:**
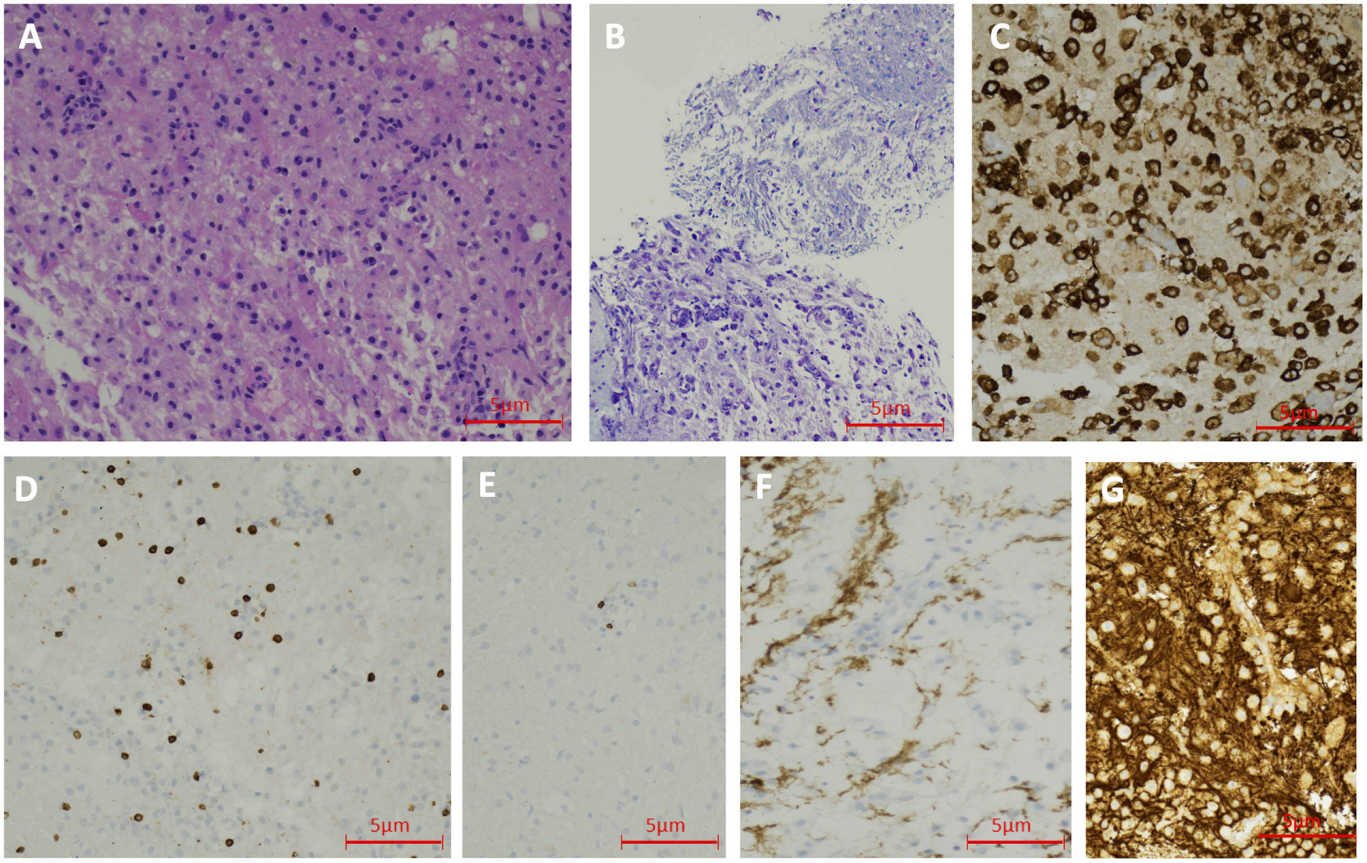
Case 2 Pathology: Hematoxylin and Eosin staining shows lymphohistocytic infiltrate in the brain tissue (200 X) **(A)**. LFB staining demonstrates demyelination (200 X) **(B)**. CD163 immunohistochemical stain shows numerous histiocytes (200 X) **(C)**. CD3 immunohistochemical stain reveals scattered T-lymphocytes (200 X) **(D)**. CD20 immunohistochemical stain demonstrates rare B-lymphocytes (200 X) **(E)**. GFAP immunohistochemical stain shows reduced astroglial density in the center of the lesion (200 X) **(F)**. Neurofilament protein reveals the preservation of axons (200 X) **(G)**.

MRI with gadolinium of the brain, cervical and thoracic cords revealed a contrast-enhancing left thalamic lesion (Figures 3A–D), and a non-enhancing, longitudinally extensive T2 hyperintensity extending from T6 to T12 levels, consistent with longitudinally extensive transverse myelitis (LETM) (Figures 3E–H). Nasopharyngeal COVID-19 PCR testing was negative. Serum spike protein antibodies were detectable at titers >250 U/mL. Serological and immunological assays were notable for elevated titers of anti-nuclear antibody (ANA) of 1:2560 (reference <1:80), and double-stranded DNA antibody (anti-DsDNA) of 158 IU/ml (reference <29). The remainder of the immune assays were normal, including serum levels of anti-aquaporin 4 (anti-AQP4) antibody utilizing enzyme-linked immunosorbent assay (ELISA) technique (completed at BN LabCorp in Burlington, NC), angiotensin converting enzyme (ACE), and C3 and C4 complement. CSF analysis demonstrated a pleocytosis of 33 cells/μL with 91% lymphocytic predominance and an elevated protein of 57 mg/dL, a negative paraneoplastic panel, undetectable anti-MOG antibody, absent oligoclonal bands, and normal IgG index. Biopsy of the left thalamic lesion demonstrated a histiocytic inflammatory process, focal perivascular lymphocytosis and reduced astroglial density at the center of the lesion (Figures 4A–G). Based on the subsequent detection of CSF anti-AQP4 antibody utilizing cell-based assay (CBA) (completed at Mayo Clinic Neuroimmunology Laboratory) with titers of 1:16 (reference <1:2) and reduced astroglial staining reflective of astroglial injury known to be associated with anti-AQP4 antibody, the diagnosis of NMOSD was entertained. The patient demonstrated a notable clinical response to an extended course of pulse IV methylprednisolone therapy followed by oral steroid tapering course, and plasmapheresis. Follow-up MRI of the brain and spinal cord at 6-months demonstrated persistent T2 hyperintense lesions in the left thalamus and thoracic cord. At 10 months follow-up, serum anti-AQP4 antibody utilizing a CBA was found to be positive, solidifying the diagnosis of NMOSD.

### Case #3: New-Onset Neuromyelitis Optica Spectrum Disorder

A 54-year-old woman with past medical history of immune thrombocytopenia purpura and family history of myasthenia gravis presented to the ED with a 2-week history of progressively ascending numbness that began 3 days following the second dose of the Moderna vaccine. Neurological findings were notable for a T3 sensory level, diffuse hyperreflexia, non-sustained ankle clonus and extensor plantar response bilaterally, without evidence of any motor weakness or gait impairment.

**Figure 5:**
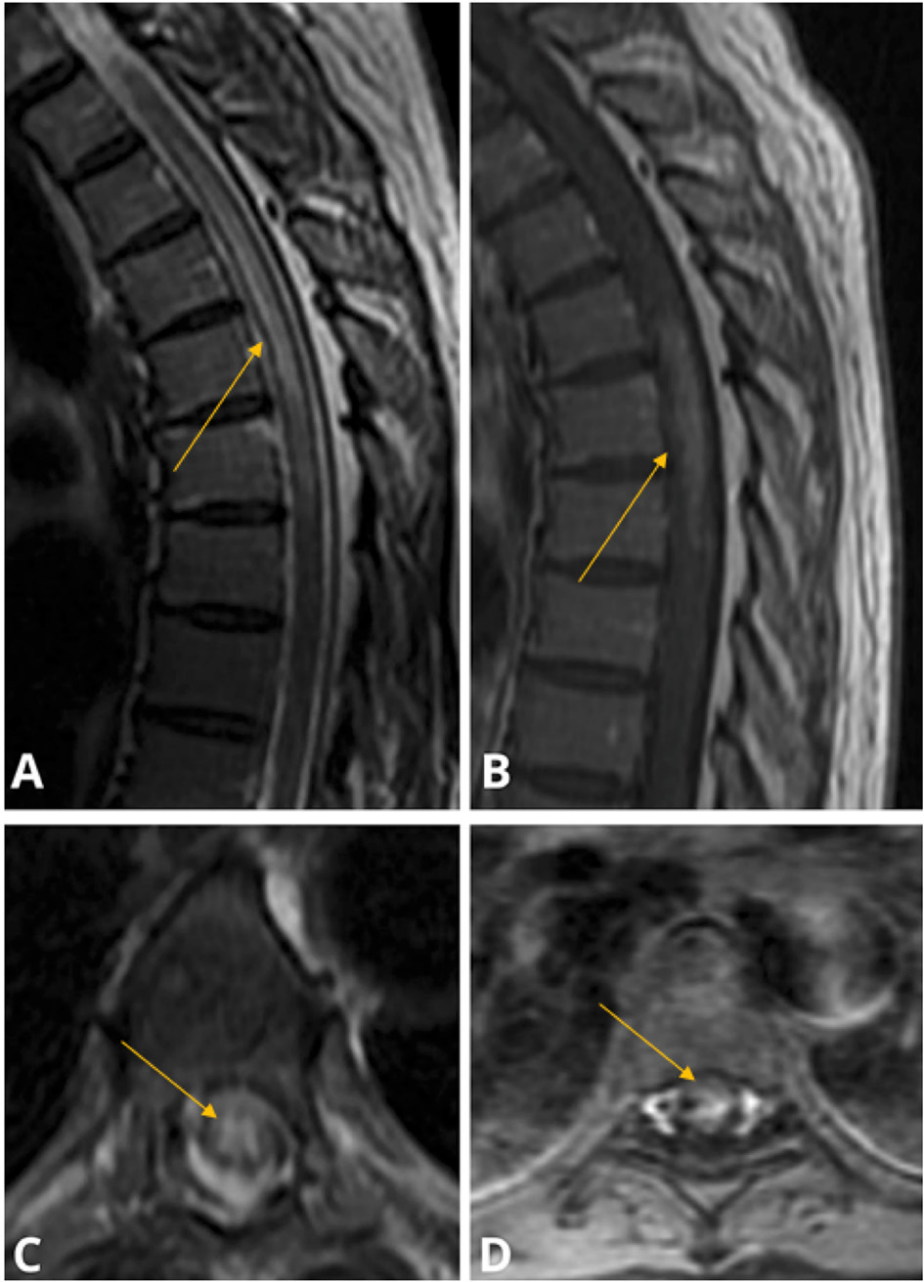
Case 3: Sagittal **(A)** and Axial **(C)** MRI T2 STIR demonstrating central gray-matter predominant hyperintensity spanning the T2–T9 vertebrae; Sagittal **(B)** and Axial **(D)** MRI T1 with gadolinium study demonstrating centrally enhancement spanning T5–T7 levels. The brain, cervical and lumbar cord were spared and thus not pictured.

Contrast-enhanced thoracic spine MRI demonstrated a longitudinally extensive T2 hyperintense lesion extending from T2 to T9 level, with gadolinium enhancement between T5 and T7 levels, consistent with LETM (Figures 5A–D). The remainder of the MRI neuroimaging, including cervical and lumbar spine, as well as brain MRI were normal. COVID-19 spike antibodies were positive (>250 U/mL) and nasopharyngeal COVID-19 PCR testing was negative. Serum and hematological assays were notable for leukopenia of 2.24 k/μL, elevated ANA titers of 1:320, and elevated serum anti-AQP4 antibody titers (utilizing ELISA) of 1,417.3 U/mL (reference 0–3.0 U/mL). The remainder of comprehensive autoimmune assays was unremarkable including ESR, CRP, c-ANCA, p-ANCA, ACE, SSA, SSB, anti-MOG and DsDNA antibodies. CSF analysis demonstrated pleocytosis of 26 cells/μL with 86% lymphocytic predominance, elevated protein of 71 mg/dL, and MBP of 27.0 ng/ml. CSF analysis demonstrated undetectable levels of anti-AQP4 (utilizing CBA) and anti-MOG antibodies, a negative paraneoplastic panel, and absent oligoclonal bands. She was treated with high dose intravenous steroids for 5 days with notable improvement of her sensory symptoms.

### Case #4: Fulminant Onset of Inflammatory Demyelinating Disease Suggestive of MS-Like Syndrome Rather Than ADEM

A 49-year-old man without prior neurological symptoms or signs presented with a 3-week history of nightly low-grade fever, diplopia, unsteady gait, and numbness of both feet. It began 4 days following the administration of the second dose of the Moderna SARS-CoV2 vaccine. Past medical history is notable for COVID-19 infection with a full recovery about 3 months before his current illness. Notable neurological findings were right leg appendicular ataxia, slightly diminished vibration sense of both lower extremities, and gait ataxia.

**Figure 6:**
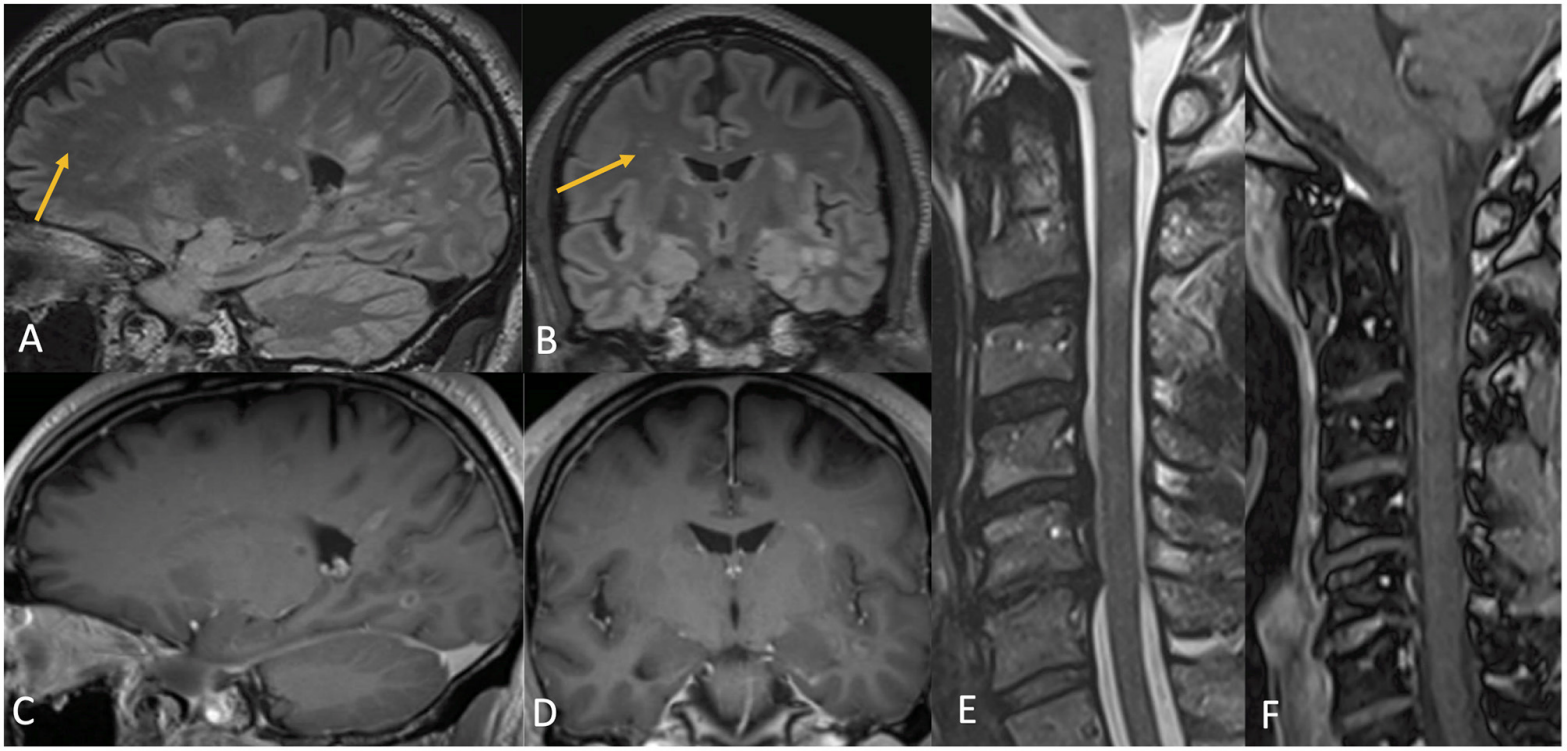
Case 4: Contrast MR brain demonstrating numerous T2 hyperintense lesions involving both the white and gray matter, many of which, but not all, had corresponding complete and incomplete ring enhancement [**(A–D)**, arrows pointing to non-enhancing lesions]. Contrast MR cervical spine demonstrates multiple abnormal T2-hyperintense signal lesions **(E)** with enhancement **(F)** of the spinal cord, most prominent at the level of the C2 and C3 vertebrae.

Contrast-enhanced brain MRI revealed numerous supratentorial and infratentorial demyelinating lesions, the vast majority of them displaying enhancement, involving white matter, thalami, and basal ganglia (Figures 6A–D). While the lesions were overall too numerous to accurately count, there were >50 contrast-enhancing brain lesions, a highly atypical finding that is consistent with a fulminant onset. There were a few old non-enhancing lesions suggestive of prior clinically-silent demyelination. A cervical spine MRI with gadolinium revealed two enhancing cervical cord lesions (Figures 6E,F). CSF analysis demonstrated a pleocytosis of 41 cells/μL with 70% lymphocytic predominance, and elevated protein of 125 mg/dL. CSF assays for oligoclonal bands and anti-MOG antibody were not performed initially due to insufficient quantity but were negative at 5-month follow-up. Nasopharyngeal SARS-CoV-2 PCR was negative. The pattern and appearance of the lesions, including those indicative of preexisting clinically-silent demyelination unaccompanied by any prior neurological symptoms, were thought to be more consistent with oligoclonal bands-negative MS variant presenting with a fulminant attack as the first clinical episode of demyelination following mRNA-based SARS-CoV-2 vaccination.

The patient demonstrated substantial clinical improvement following treatment with a 5-day course of pulse IV methylprednisolone therapy with a dose of one gram/day, followed by oral steroid tapering course. At 10-month follow-up visit, the patient remained in clinical remission with brain MRI showing persistent T2-hyperintense lesions with complete resolution of gadolinium enhancement and lack of new demyelinating lesions.

### Case #5: Meningoencephalitis of Suspected Immune Origin: Possible Seronegative Autoimmune Encephalitis

A 27-year-old woman presented to the ED with a 2-day history of acute-onset confusion and anxiety of unclear etiology. It started 6 days following the first dose administration of Pfizer-BioNTech SARS-CoV-2 vaccine that resulted in transient headache and fatigue. She had no prior neurological illness. She had a fever of 100.4° F without nuchal rigidity. Neurological examination revealed psychomotor agitation, dysfluent speech with paraphasic errors, and difficulty with writing. Serological assays were notable for a mildly elevated ESR to 21 mm/h (reference range 0–15 mm/h). There was no leukocytosis. Antibodies against COVID-19 Spike were positive (>250 U/ml) and nasopharyngeal COVID-19 PCR test was negative. Thyroid functions were normal. A Mayo Clinic serum autoimmune encephalitis panel (ID: ENS2) was negative. CSF analysis was notable for pleocytosis of 19 WBCs with 84% lymphocytic predominance, and normal protein and glucose. CSF infectious workup was negative, including bacterial and fungal cultures as well as viral PCR panel (which includes HSV-1, HSV-2, HHV-6, CMV, VZV, Enterovirus, Parechovirus). Anti-NMDA receptor antibody was not detected in the CSF; the remainder of CSF autoimmune encephalitis panel was not completed due to insufficient CSF quantity. CSF Oligoclonal bands were absent and IgG index was normal. Contrast enhanced brain MRI showed no abnormalities. Electroencephalography (EEG) showed mild generalized slowing without epileptiform abnormalities. She was started on a 5-day course of pulse IV methylprednisolone with a dose of one gram/day for presumed autoimmune meningo-encephalitis with a significant clinical improvement. She was subsequently discharged on an oral tapering course of steroid with a steady improvement. She achieved a full recovery in about 1 month after discharge; thus, no further imaging of the brain or body was performed.

## Discussion

We report five cases of clinically new-onset CNS events of suspected immune origin (ADEM; *n* = 1, NMOSD; *n* = 2, MS-like syndome; *n* = 1, meningoencephalitis; *n* = 1) that were temporally associated with mRNA-based SARS-CoV-2 vaccination in a single health system. All cases have no antecedent clinically evident CNS autoimmunity. In one case (Case 4), brain MRI showed potentially older, but clinically silent, small non-enhancing demyelinating lesions. All cases, except Case 1 of fatal fulminant ADEM, were responsive to immune therapy, including steroid and/or plasmapheresis (Table 1).

**Table 1:** Cases overview.

	**Case #1**	**Case #2**	**Case #3**	**Case #4**	**Case #5**
Age (years)	81	63	54	49	27
Sex	Male	Woman	Woman	Man	Woman
Pre-existing neurological illness	Absent	Absent	Absent	Absent	Absent
Pre-existing history of autoimmune disease	Absent	Absent	Yes; ITP	Absent	Absent
Vaccine	Moderna (mRNA-1,273)	Pfizer-BioNTech (BNT162b2)	Moderna (mRNA-1,273)	Moderna (mRNA-1,273)	Pfizer-BioNTech (BNT162b2)
Onset relative to vaccine dose	13 days after 1st dose	7 days after 1st dose	3 days after 2nd dose	4 days after 2nd dose	6 days after 1st dose
New neurological symptoms	Severe confusion	Weakness, urinary retention	Ascending numbness	Diplopia, unsteady gait, numbness	Confusion, anxiety, headache
CSF WBC count (cells/uL) (ref 0–5 cells/uL)	3, 11, 69	33	26	41, 3^∧^	19
CSF Protein count (mg/dL) (ref 15–45mg/dL)	45, 52, 45	57	71	125, 166	43
Brain involvement	Brainstem, Thalamus	Thalamus	Spared	Diffuse	Spared
Spinal cord involvement	Unknown	LETM; thoracic; central	LETM; thoracic; central	Multifocal; short segment; cervical	Unknown
Anti-AQP4 CSF ab	Negative	Positive	Negative	Unknown	Unknown
Anti-AQP4 serum ab	Negative	Negative, positive	Positive	Negative	Unknown
Anti-MOG CSF ab	Negative	Negative	Negative	Negative	Unknown
Anti-MOG Serum ab	Unknown	Negative	Unknown	Negative	Unknown
Treatment (No. of days)	IVMP (7), IVIG (5), PLEX (4)	IVMP (5), PLEX (7)	IVMP (5)	IVMP (5)	IVMP (5)
Response to treatment	Expired on hospital day 26	Improvement	Improvement	Improvement	Improvement
Final diagnosis	ADEM	NMOSD (new onset)	NMOSD (new onset)	Fulminant MS (new onset)	Meningoencephalitis of immune orgin

*LETM, Longitudinally Extensive Transverse Myelitis; AQP4, Aquaporin-4; MOG, Myelin Oligodendroglycoprotein; IVMP, intravenous Methylprednisolone; IVIG, intravenous immunoglobulins; PLEX, plasmapheresis. ^∧^CSF study performed at 5-month follow-up*.

The first case highlights a patient with rapid decline into coma within a few hours of arrival, despite having normal CSF analysis and minimal involvement of the brainstem on initial brain MRI. Benign CSF findings are not atypical in ADEM, with normal CSF leukocyte counts and protein levels reported in about half of patients (16). MRI abnormalities may be minimal or absent during the first 2 weeks of ADEM presentation (17). The rapid clinical decline can be attributed to increased brainstem involvement that became more evident on subsequent neuroimaging. ADEM subgroups presenting with rapid decline in consciousness are associated with higher mortality rates (18).

The second and third cases involve patients who developed LETM within 1 week of vaccine administration and also had positive anti-AQP4 antibodies. In the second case, the initial anti-AQP4 antibody test was positive in the CSF (utilizing CBA) but negative in the serum (utilizing ELISA), with positive repeat serological testing (utilizing CBA) 10 months later. Atlhough the initial combination of anti-AQP4 antibody results in this case—CSF positive but serum negative—is highly atypical of NMOSD (19–22), the initial negative serum test was likely a false negative due to the lesser sensitivity and specificity of the ELISA test compared to the CBA modality (23). While the cerebrum was spared in Case 3, the thalamus was involved in Case 2, a structure that can be involved in NMOSD (24). Both cases involved the centrally located gray-matter of the thoracic spinal cord, a common radiographic finding in NMOSD (“H-Sign”) (24).

Of note, both patients are potentially at greater risk of developing autoimmunity (elevated ANA titers in both cases, and positive personal and family history of autoimmune disease in Case 3). NMOSD has been shown to be associated with other coexisting systemic autoimmune disorders such as autoimmune hematological disorders (25). Similarly, one case of new-onset NMOSD was recently reported following SARS-CoV-2 vaccination (11). While viral infections are known to serve as immunological triggers of CNS autoimmunity (26), the evidence associating vaccines with short-term increase in new onset or recurrence of CNS inflammatory and immunological events among patients with underlying autoimmune disease is mixed (26–29). Currently, therefore, most vaccines are generally recommended for this patient population. The current CDC guidelines permit SAR-CoV-2 vaccination for patients with autoimmune conditions but do admit to the scarcity of the currently available safety data, despite enrollment of this population in the various vaccine clinical trials (30).

The fourth case involves a patient who had numerous multifocal gadolinium-enhancing T2 lesions throughout the cerebrum and cervical spinal cord. ADEM diagnosis was considered in this 49-year-old man given the enhancement of the vast number of T2 hyperintense lesions, the lack of CSF oligoclonal bands, and the complete clinico-neuroradiological stability off preventative therapy at 10-month follow-up. However, the diagnosis of oligoclonal bands-negative MS-like syndome presenting with a fulminant attack as the first clinically-evident demyelinating episode, which is temporally associated with mRNA-based SARS-CoV-2 vaccination, is thought to provide a “better explanation” for the patient's presentation. This is supported by the presence of a few small non-enhancing lesions suggestive of a prior clinically-silent mild demyelination. Accordingly, the patient appeared to meet 2017 McDonald criteria for relapsing remitting MS based on one clinical episode and MRI findings demonstrating lesions dissemination in time and space that lack a better explanation.

This case adds to the recently published cases of steroid responsive MS exacerbations (11), and first-clinical manifestations of MS (11, 12, 14), following the mRNA-based SARS-CoV-2 vaccines. In a recent study of 555 recipients of the Pfizer vaccine with a known MS diagnosis, there was a 2% relapse rate among vaccinated participants, which was comparable to that observed among non-vaccinated individuals (13). Further, except for a small study that linked yellow fever to the exacerbation of MS relapses (4, 5), vaccines in general have not been previously linked to MS relapses. Indeed, a recent systematic review found no change in the risk of developing MS or increased risk of MS relapses after many commonly administered vaccines (29). In general, most vaccines—including mRNA SARS-CoV-2 vaccines—are currently recommended for patients with MS (30–32). However, further prospective studies are needed to confirm this short-term association with steroid responsive clinical relapse (11).

The fifth case involves a patient who presented with an acute encephalopathy associated with rapid cognitive decline within a week of vaccine administration. The diagnostic workup was notable for normal neuroimaging and CSF lymphocytic pleocytosis unexplained by infections or systemic autoimmunity. The rapid decline in mental status and cognitive functions, including working memory, in association with CSF inflammatory response together with the clinically meaningful response to steroids, suggest possible CNS autoimmunity such as seronegative autoimmune encephalitis (33–35). This is consistent with the rapidly increasing proportion of patients with encephalitis thought to be of immune origin despite undetectable neuronal surface autoantibodies in serum and CSF (15, 33–35), and absent structural abnormalities on neuroimaging studies (35). The data linking vaccines to autoimmune encephalitis are very scarce and largely limited to sporadic cases of yellow fever vaccine (36, 37). Therefore, it remains unclear whether this presentation is a coincidental event that occurred following, or an adverse event linked to, vaccination. Across the five cases, we enrolled patients who developed CNS inflammatory symptoms within 2 weeks of either vaccine dose, in keeping with the published reports of post-vaccination autoimmunity (38).

The following is a summary of the reported adverse CNS events of suspected immune origin during clinical trials: there are four different vaccine platforms being used to deliver the SARS-CoV-2 vaccine: inactivated, viral vector, protein subunit, and nucleic acid-based vaccines (39). The nucleic acid-based vaccines are the most recent development with many phase-1 clinical trials demonstrating their safety (40–42). Aside from three reported cases of Bell's Palsy in the clinical trial for the Moderna-developed mRNA-1273 vaccine, there were no other neurological side effects in any of the 14,134 vaccinated patients (43). Similarly, there were no reported neurological adverse events, including those of suggested immune origin, among the 21,720 vaccinated patients in the Pfizer-BioNTech phase 2/3 trial (44). Three cases of transverse myelitis were reported in the phase 3 clinical trial for the AstraZeneca-developed vector-based vaccine; two of these cases were later deemed to be unlikely related to the vaccine (45). Using a similar vaccine platform, the clinical trial for the Johnson and Johnson Jansen Ad26.COV2.S vaccine reported one case of Guillian Barre Syndrome (GBS) and one case of facial palsy (39). However, there was also one case of GBS in the placebo group, which is likely in line with background incidence rates in the general population (46). As of March 2, 2021, the database from Centers for Disease Control (CDC) Vaccine Adverse Event Reporting System (VAERS) revealed only nine cases of transverse myelitis and six cases of ADEM following administration of 51,755,447 dosages of the COVID-19 vaccines (47). Since the VAERS database is based on passive surveillance, it has several limitations, including reporting bias and errors.

It should be emphasized that post-vaccination short-term increase in the rate of inflammatory and autoimmune CNS pathologies has been associated with a wide range of vaccines using various platforms (38). This increase is not uniquely linked to specific mechanism or platform (48). However, given the suspected strong immunogenic properties of nucleic acids, which are partially attributed to their abilities to activate transmembrane Toll-like receptors (TLR) (49), careful assessment should be given when considering mRNA-based vaccines for those potentially at greater risk of vaccine-related CNS events of immunological origin (50–52). Such individuals include those with underlying immune-mediated disorders associated with aberrantly upregulated immunological and inflammatory cascades as well as those with genetically pre-determined reduced clearance of nucleic acids that may potentially increase the risk of nucleic acids-induced immunogenicity (49, 50). Furthermore, like other viruses, prior SARS-CoV-2-infection can serve as an immunological trigger through several potential mechanisms, including suggested molecular mimicry involving cross-reactive autoantibodies targeting SARS-CoV-2 antigens (53). This can aberrantly upregulate pro-inflammatory factors, which in turn may increase blood-barrier permeability and vaccine-related CNS autoimmunity propensity (51).

Overall, adverse neuroinflammatory and immunological events across all the major clinical trials that assessed the safety profile of the various SARS-CoV-2 vaccines were reportedly very rare, with zero reported cases of CNS inflammatory/autoimmune disorders in the mRNA-based vaccine trials (43, 44). The reported temporal association between inoculation with mRNA SARS-CoV-2 vaccine and the development of various CNS disorders of suspected inflammatory and immune origin is consistent with the clinical anecdotes associating other vaccines with short-term increase in CNS autoimmunity such as inflammatory demyelinating disease (38). Our findings suggest that the occurrence of post-mRNA SARS-CoV-2 vaccination CNS autoimmunity, new-onset or clinical relapse, might be more prevalent among those with pre-existing CNS autoimmunity or increased propensity for systemic autoimmune disorders. However, this short-term association should be interpreted with great caution. It neither proves a causal link nor implies a potential long-term risk increase in post-vaccination CNS autoimmunity. Clinicians should be aware of this potential association as early immune therapies may likely reverse these observed immunological events. Limitations to this study include the small sample size, given the brief period since SARS-CoV-2 vaccines have been available, data limited to a single hospital system, as well as the retrospective nature of the study.

## Conclusions

To our knowledge, these are among the emerging cases of CNS adverse events of suggested immunological or inflammatory origin that occurred within 2 weeks of inoculation with an mRNA-based SARS-CoV-2 vaccine. Although our report neither proves a mechanistic link nor implies a potential long-term increase in post-vaccination CNS autoimmunity, the finding of five cases at a single health system suggests that these immunogenic events are potentially more common than currently known, particularly among subgroups of patients such as those with pre-existing CNS or systemic autoimmune diseases. The limited number of similar reports and the significant observed response to immune therapies suggest that this short-term association is likely rare and often reversible. Given the novelty of the mRNA-based vaccine platform and the suspected greater immunogenic potency of nucleic acids, large prospective epidemiological studies to fully assess the potential association between these vaccines and CNS autoimmunity are warranted, particularly among those with underlying CNS or systemic autoimmune disorders.

## Data Availability Statement

The raw data supporting the conclusions of this article will be made available by the authors, without undue reservation.

## Ethics Statement

The studies involving human participants were reviewed and approved by Feinstein Institute for Medical Research IRB (approval # 20-0600). The patients/participants provided their written informed consent to participate in this study. Written informed consent was obtained from the individual(s) for the publication of any potentially identifiable images or data included in this article.

## Author Contributions

ABal, ABab, MK, JL, and AH contributed to conceptualization, literature search, writing—original draft, writing—review and editing, and verified underlying data. NH, GW, SS, and FC contributed to writing—review and editing, validation, and supervision. SN contributed to writing—review and editing, validation, supervision, and verified underlying data. All authors contributed to the article and approved the submitted version.

## Conflict of Interest

The authors declare that the research was conducted in the absence of any commercial or financial relationships that could be construed as a potential conflict of interest.

## Publisher's Note

All claims expressed in this article are solely those of the authors and do not necessarily represent those of their affiliated organizations, or those of the publisher, the editors and the reviewers. Any product that may be evaluated in this article, or claim that may be made by its manufacturer, is not guaranteed or endorsed by the publisher.
